# Environmental injustice in the West Bank: a mixed-methods assessment using SDG-based indicators

**DOI:** 10.1186/s12889-026-28228-w

**Published:** 2026-07-06

**Authors:** Nidal Mohammad Nassar, Hamzeh Al Zabadi, Zohra Lili Chabaane

**Affiliations:** 1 University of Carthage, National Agronomic Institute of Tunisia, Laboratory GREEN-TEAM LR17AGR01, 43 Avenue Charles Nicolle, Tunis Mahrajène, 1082 Tunisia; 2 Institution of Agricultural Research and Higher Education (IRESA), National Agronomic Institute of Tunisia, Laboratory GREEN-TEAM LR17AGR01, 43 Avenue Charles Nicolle, Tunis Mahrajène, 1082 Tunisia; 3 Public Health Department, Faculty of Medicine and Allied Medical Sciences, An-Najah National University, Nablus, Palestine

**Keywords:** Environmental justice, Environmental inequalities, Sustainable Development Goals (SDGs), Sustainable development, Environmental governance, Mixed-methods, West Bank, Occupied Palestinian Territory

## Abstract

This study examines environmental justice in the West Bank, Palestine, within the broader framework of sustainable development. Building on environmental justice theory, which emphasizes distributive, procedural, and recognition dimensions, the research investigates perceptions of environmental justice and environmental inequalities within a context characterized by complex governance and resource constraints.

A mixed-methods approach was employed, combining quantitative data from a structured questionnaire (n = 629) with qualitative responses obtained through open-ended questions included in the survey instrument. The survey assessed perceptions across sixteen indicators aligned with the Sustainable Development Goals (SDGs), covering key domains such as economic conditions, inequalities, sustainable cities, environmental management, climate action, biodiversity, institutional performance, and global partnerships.

The findings indicate high levels of perceived environmental justice concerns across all indicators. Statistical analysis revealed no significant differences across gender, place of residence, or administrative division for most indicators, suggesting broadly similar perceptions among respondents. However, significant differences emerged for the Global Partnership for Sustainable Development indicator across gender and administrative divisions.

The qualitative findings highlight the perceived importance of governance-related challenges, institutional limitations, infrastructure constraints, and resource accessibility in shaping environmental justice concerns. These findings provide additional context for interpreting the quantitative results and contribute to a more comprehensive understanding of environmental justice perceptions in the West Bank.

The study concludes that environmental justice concerns in the West Bank extend across multiple environmental, social, economic, governance, and development-related dimensions. The research contributes to the environmental justice literature by integrating SDG-based indicators within a mixed-methods framework and by providing evidence from a politically and institutionally constrained context.

**Supplementary Information:**

The online version contains supplementary material available at https://doi.org/10.1186/s12889-026-28228-w.

## Introduction

Environmental justice (EJ) has emerged as a critical framework for understanding inequalities in environmental quality, access to resources, and participation in decision-making processes. As conceptualized by scholars such as Schlosberg [25] and Walker [29], environmental justice extends beyond the distribution of environmental risks and benefits to include procedural fairness and the recognition of marginalized communities. These three interrelated dimensions—distributive, procedural, and recognition justice—form the foundation for analyzing environmental inequalities in diverse contexts.

Historically, the environmental justice movement gained prominence in the United States during the 1980 s, where grassroots mobilization exposed the disproportionate placement of environmental hazards in marginalized communities [26]. Since then, the concept has evolved globally, taking different forms depending on regional contexts. In Europe, environmental justice has been shaped through human rights frameworks and institutional mechanisms such as the Aarhus Convention [28], adopted in (1998), which emphasizes access to information, participation, and justice [20]. In Latin America and Sub-Saharan Africa, environmental justice is closely linked to issues of industrialization, resource extraction, and the struggles of marginalized communities, particularly Indigenous populations, to protect their environments and livelihoods [2, 17].

Across these contexts, environmental justice is increasingly understood as a multidimensional issue embedded within broader socio-economic and political structures. Studies have shown that environmental policies and development processes often produce uneven outcomes, disproportionately affecting low-income and vulnerable populations [9]. Barriers to participation, including technical, economic, and institutional constraints, further limit the ability of marginalized groups to influence environmental governance [6, 7]. Moreover, environmental injustice is frequently intensified in contexts characterized by social vulnerability and limited adaptive capacity [15].

Within the framework of sustainable development, environmental justice has become central to achieving equitable and inclusive outcomes. The Sustainable Development Goals (SDGs) emphasize reducing inequalities, promoting sustainable cities, ensuring environmental protection, and strengthening institutions. However, recent scholarship highlights that the implementation of these goals is highly contingent on political and structural conditions, particularly in regions affected by conflict or external control.

The Palestinian context, and specifically the West Bank, provides a critical case for examining environmental justice under such conditions. Existing studies indicate that environmental inequalities in the region are closely linked to the Israeli occupation, which imposes restrictions on land use, infrastructure development, and resource management [23, 31]. These constraints have resulted in significant disparities in access to water, waste management systems, and environmental services, while contributing to pollution and environmental degradation.

Furthermore, research has shown that environmental challenges in Palestine are compounded by institutional, financial, and technical limitations, as well as fragmented governance structures [3, 5]. Waste management, in particular, represents a critical issue, as inadequate infrastructure and regulatory frameworks lead to environmental contamination and health risks [22, 27] These environmental conditions are closely linked to environmental health outcomes, as they influence exposure to pollutants, water quality, sanitation, and overall population well-being. In some cases, the West Bank has been used as a dumping ground for hazardous waste, exacerbating environmental injustice and increasing the vulnerability of Palestinian communities [13].

These conditions reflect a broader pattern in which environmental injustice is not only a result of unequal distribution but is also shaped by structural and political dynamics that limit local agency and institutional capacity. Despite the growing body of literature on environmental conditions in Palestine, there remains a lack of comprehensive studies that integrate multiple dimensions of environmental justice within a unified analytical framework.

Understanding environmental justice in this context is essential not only for addressing inequality but also for identifying potential environmental health risks associated with unequal exposure to environmental hazards.

This study addresses this gap by examining environmental justice in the West Bank through sixteen indicators aligned with the SDGs, using a mixed-methods approach that combines quantitative analysis of citizens’ perceptions with qualitative insights from interviews. By analyzing variations across gender, place of residence, and administrative divisions, the study aims to provide a nuanced understanding of how environmental justice is experienced across different segments of society.

In doing so, the research contributes to the broader environmental justice literature by demonstrating how global frameworks such as the SDGs interact with local political realities, and by highlighting the need for context-sensitive approaches to sustainable development in conflict-affected regions.

## Research questions and hypotheses

Building upon the environmental justice literature, Sustainable Development Goals (SDGs) framework, and the unique political and environmental context of the West Bank, this study seeks to address the following research questions (RQ):

- RQ1: To what extent do residents of the West Bank perceive environmental injustice across SDG-based environmental, social, economic, and governance dimensions?
- RQ2: Do perceptions of environmental injustice differ according to gender, place of residence (city, village, or refugee camp), and administrative region (North, Middle, and South West Bank)?
- RQ3: How do qualitative interviews explain and contextualize the quantitative patterns identified through the survey findings?
- RQ4: What are the perceived implications of environmental injustice for environmental conditions, environmental health, and sustainable development in the West Bank?

Based on the existing literature and the objectives of the study, the following hypotheses were examined:

- H1: Respondents perceive high levels of environmental injustice across SDG-based dimensions in the West Bank.
- H2: Perceptions of environmental injustice differ according to gender.
- H3: Perceptions of environmental injustice differ according to place of residence.
- H4: Perceptions of environmental injustice differ according to administrative region.

The integration of quantitative and qualitative evidence was intended to provide a more comprehensive understanding of environmental justice perceptions and their underlying contextual drivers.

### Study contribution

This study contributes to the environmental justice and sustainable development literature in several important ways.

First, it develops and applies an SDG-aligned framework for assessing environmental justice perceptions across sixteen dimensions reflecting environmental, social, economic, governance, and development-related concerns. By linking environmental justice principles with the Sustainable Development Goals, the study provides a structured approach for evaluating environmental inequalities within a broader sustainability context.

Second, the study extends environmental justice research to a politically constrained and conflict-affected setting. While environmental justice scholarship has traditionally focused on industrialized countries and marginalized communities within national contexts, relatively few studies have examined environmental justice under conditions of prolonged political conflict, territorial fragmentation, and institutional constraints.

Third, the study integrates quantitative survey data from 629 respondents with qualitative interview evidence to provide both breadth and contextual depth. This mixed-methods approach enables a more comprehensive understanding of how environmental injustice is perceived and experienced across different social and geographical groups.

Fourth, the study contributes empirical evidence from the West Bank, a region that remains underrepresented in international environmental justice literature. The findings provide insights into how environmental governance challenges, resource inequalities, and development constraints interact within a unique socio-political context.

Finally, the study contributes to ongoing discussions regarding the implementation of the Sustainable Development Goals in contexts characterized by environmental vulnerability, governance challenges, and restricted access to natural resources. In doing so, it offers a framework that may inform future environmental justice assessments in other resource-constrained and politically complex settings.

## Literature review

### Conceptual foundations of environmental justice

Environmental justice (EJ) has evolved into a multidimensional analytical framework concerned with inequalities in environmental quality, access to resources, and participation in decision-making processes. As highlighted by Schlosberg [25] and Walker [29], environmental justice is typically conceptualized through three interrelated dimensions: distributive justice, procedural justice, and recognition justice.

Distributive justice focuses on the unequal allocation of environmental risks and benefits across populations, while procedural justice emphasizes fair and inclusive participation in environmental governance [7]. Recognition justice, in turn, addresses the marginalization of communities’ identities, knowledge, and rights, particularly among vulnerable and Indigenous populations [30]. Together, these dimensions underscore that environmental inequalities are not merely technical issues, but are socially and politically constructed, as environmental resources and risks are unevenly distributed and unequally accessible.

### Global evolution of environmental justice

The emergence of environmental justice as a field of study is closely tied to socio-political movements. In the United States, the EJ movement gained momentum during the 1980 s, when grassroots protests exposed the disproportionate siting of hazardous waste facilities in African American communities [26]. In Europe, environmental justice developed within a human rights framework, particularly through institutional mechanisms such as the Aarhus Convention (1998), which formalized rights related to access to environmental information, public participation, and legal recourse [20]. Similarly, in Latin America, environmental justice has been shaped by rapid industrialization and urbanization, as well as the struggles of Indigenous communities to defend their natural resources and environmental rights [10, 17].

In Sub-Saharan Africa, environmental justice is closely linked to development challenges, including extractive industries and the dependence of rural populations on natural resources [2]. In South Africa, environmental justice activism emerged within the anti-apartheid movement and later became embedded in constitutional frameworks [19].

Across these diverse contexts, environmental justice has consistently been associated with broader socio-economic inequalities and power imbalances, reinforcing its relevance as a global analytical framework.

### Environmental justice, inequality, and development

A growing body of literature highlights the relationship between environmental justice and development processes. Environmental policies and economic transitions often generate uneven benefits and costs, disproportionately affecting low-income and marginalized communities [9]. These impacts may manifest spatially through processes such as environmental gentrification, displacement, and unequal access to environmental services.

Moreover, barriers to procedural justice—such as limited access to information, technical complexity, and socio-economic constraints—can restrict meaningful participation in environmental governance [6]. As a result, vulnerable communities often face reduced adaptive capacity and increased exposure to environmental risks [15]. Such exposure is often associated with adverse environmental health outcomes, particularly among vulnerable populations.

These dynamics underscore the importance of integrating environmental justice into sustainable development frameworks, particularly to ensure that development transitions do not exacerbate existing inequalities.

### Environmental justice in the Palestinian context

In the Palestinian context, environmental justice is deeply intertwined with political and structural constraints. Studies have demonstrated that the Israeli occupation significantly shapes environmental conditions in the West Bank by restricting land use, infrastructure development, and resource management [23].

For example, limitations on the construction of sewage treatment facilities have contributed to widespread pollution of land and groundwater, which constitute the primary sources of drinking water in the region. Similarly, restrictions on land classified as “nature reserves” limit Palestinian access to resources and result in substantial economic losses [31].

Human Rights Watch [14] further documents how land use practices, including the conversion of valleys and mountains into recreational areas for settlers, exacerbate spatial inequalities while restricting Palestinian mobility and access.

Recent Palestinian scholarship has similarly emphasized the interconnected relationship between environmental challenges, resource governance, and sustainable development. Alayan and Al-Natour [1], in their analysis of water scarcity framings in Palestinian educational materials, demonstrated that water-related challenges are frequently understood within broader political, environmental, and developmental contexts. Their findings highlight the central role of resource access, governance structures, and sustainability considerations in shaping environmental realities in Palestine, reinforcing the relevance of environmental justice perspectives in understanding environmental inequalities within the West Bank.

### Waste management and environmental inequality

Solid waste management represents one of the most critical environmental challenges in Palestine. Rapid population growth, urban expansion, and inadequate infrastructure have led to the accumulation of waste containing hazardous substances, posing risks to human health and the environment [16, 18, 22, 27].

These risks include increased incidence of respiratory diseases, waterborne infections, and long-term exposure to environmental contaminants [12].

In the West Bank, these challenges are intensified by restrictions imposed by the Israeli occupation, which hinder the establishment of sanitary landfills and effective waste management systems [3]. The resulting lack of infrastructure exacerbates environmental degradation and negatively affects the daily lives of Palestinian communities [5]. Moreover, the issue of waste management is shaped by a complex interaction of technical, institutional, legal, financial, and political factors, reflecting the broader structural constraints faced by Palestinian society [3, 21]. Recent reports also highlight the use of the West Bank as a dumping ground for Israeli waste, including hazardous materials, which imposes disproportionate environmental and health burdens on Palestinian communities [13]. These practices extend beyond conventional environmental inequalities, reflecting a form of structurally embedded environmental injustice.

### Institutional constraints and environmental governance

Environmental governance in Palestine is further constrained by institutional limitations, including weak regulatory frameworks, insufficient financial resources, and limited technical capacity [3, 4]. The absence of effective monitoring and enforcement mechanisms undermines the ability of institutions to manage environmental challenges and ensure equitable service provision.

Additionally, fragmented governance structures and restrictions on movement and planning reduce coordination across regions, further complicating environmental management efforts. This limitation can exacerbate environmental health risks by reducing the capacity to monitor, regulate, and mitigate environmental hazards. These conditions hinder the realization of environmental justice, as communities are unable to access or utilize resources equitably.

### Environmental justice as a structural condition

Taken together, the literature suggests that environmental injustice in the Palestinian context cannot be understood solely in terms of unequal distribution of environmental risks. Rather, it reflects a broader system of structural inequality, where environmental outcomes are shaped by political, institutional, and socio-economic factors.

This perspective aligns with broader findings in environmental justice research, which indicate that marginalized communities often bear disproportionate environmental burdens due to limited influence over decision-making processes [8, 11, 24].

This interpretation is also consistent with recent Palestinian research emphasizing that environmental and resource-related challenges, particularly those associated with water scarcity, are closely linked to governance, sustainability, and broader socio-political conditions [1].

In the West Bank, these dynamics are intensified by the interaction between environmental constraints and political conditions, contributing to patterns of environmental injustice that appear to be widespread across different geographical and social contexts. This interpretation is consistent with the literature on structural environmental inequalities, while recognizing that the present study primarily examines citizens’ perceptions rather than directly measuring objective environmental conditions.

Based on the reviewed literature and the theoretical foundations of environmental justice, this study proposes a conceptual framework that illustrates how environmental injustice may be shaped and perceived within the West Bank context. The framework links political and institutional conditions with environmental justice dimensions and SDG-based indicators to support the assessment of perceived environmental justice outcomes. The framework links the broader political context with structural constraints, environmental justice dimensions, and SDG-based indicators, ultimately leading to perceived environmental justice outcomes.

**Fig. 1:**
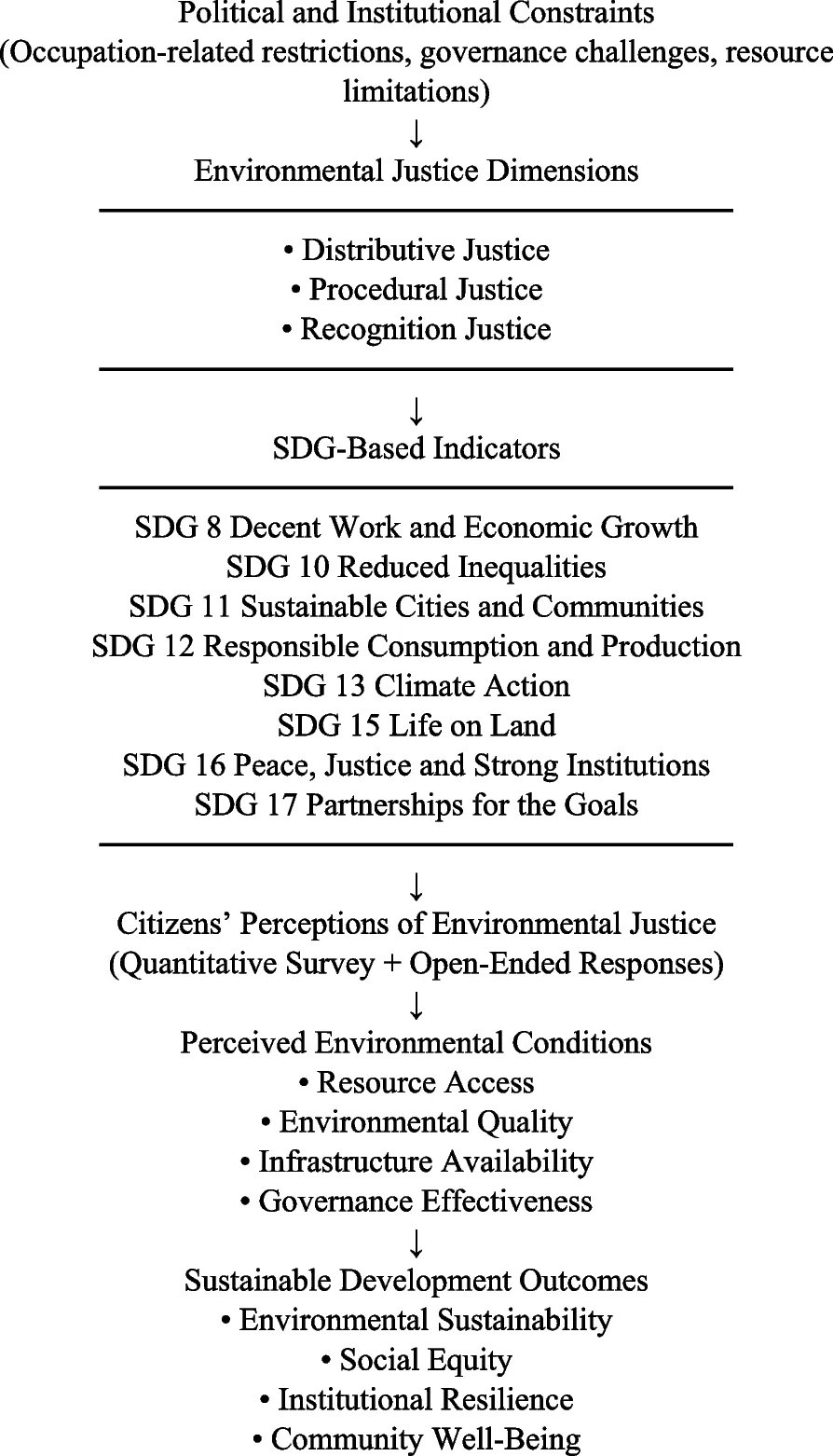
Conceptual Framework Illustrating the Relationships Between Political Constraints, Environmental Justice Dimensions, SDG-Based Indicators, and Sustainable Development Outcomes in the West Bank

Figure 1 illustrates the conceptual framework underpinning this study. The framework presents a hierarchical model in which political and institutional constraints generate structural conditions that influence environmental justice in the West Bank. These influences operate through the three core dimensions of environmental justice—distributive justice, procedural justice, and recognition justice—which provide the theoretical foundation for the study.

These dimensions are operationalized through SDG-based indicators that capture environmental, social, economic, governance, and development-related aspects of environmental justice. The indicators serve as measurement tools for assessing citizens’ perceptions regarding environmental inequalities, governance challenges, resource accessibility, and sustainable development constraints.

Quantitative survey responses and qualitative open-ended comments provide the empirical basis for evaluating perceived environmental justice conditions. The framework further recognizes that environmental inequalities may have implications for environmental conditions, access to environmental resources and services, and broader sustainable development outcomes. Accordingly, the study examines environmental justice within an integrated sustainability framework that links political context, environmental governance, SDG-based indicators, and perceived environmental outcomes.

## Methodology

### Research design

This study adopts a mixed-methods research design to provide a comprehensive understanding of environmental justice in the West Bank, Palestine. The integration of quantitative and qualitative approaches allows for both the measurement of general patterns and the exploration of in-depth lived experiences.

The quantitative component is based on a structured questionnaire developed specifically for this study (see Supplementary File 1), designed to assess citizens’ perceptions of environmental justice across multiple domains aligned with the Sustainable Development Goals (SDGs). The questionnaire was anonymous, and no personal identifying information was collected.

The qualitative component complements this analysis through open-ended qualitative responses collected as part of the survey instrument, providing contextual insights into the structural and institutional factors shaping these perceptions.

This design enhances the validity of the findings by enabling triangulation, where statistical results are interpreted alongside qualitative narratives.

### Study area and population

The study was conducted in the West Bank, Palestine, which is characterized by a fragmented geopolitical landscape and varying administrative divisions. The research covers three main regions: Northern West Bank, Central West Bank and Southern West Bank.

In addition, the study considers different types of residential settings: Cities, Villages and Refugee camps.

The study population includes Palestinian residents living in these areas during the year 2024, representing diverse socio-spatial and demographic conditions.

### Sampling and data collection

#### Quantitative component

A total of (629) respondents participated in the survey. The sample was distributed across regions (north, central, south) and residential categories (cities, villages, and refugee camps), ensuring representation of different spatial and social contexts.

Data were collected using a structured questionnaire administered to participants. The instrument was designed to capture perceptions related to environmental justice and the role of the Israeli occupation and its military policies in influencing environmental, economic, and social conditions.

#### Qualitative component

To complement the quantitative survey findings, the questionnaire included an open-ended section that allowed respondents to provide additional comments and explanations regarding environmental justice conditions in the West Bank.

A total of 100 respondents provided qualitative responses to the open-ended questions. Participants were drawn from the northern, central, and southern regions of the West Bank and represented diverse residential and social backgrounds. All respondents were aged 18 years or older.

The qualitative responses were collected electronically as part of the same survey instrument and were analyzed using thematic analysis. Responses were reviewed, coded, and grouped into recurring themes related to environmental inequalities, environmental governance, access to environmental resources and services, sustainable development challenges, and perceived drivers of environmental injustice.

The qualitative findings were used to provide contextual interpretation of the quantitative results and to enhance understanding of the patterns identified through the statistical analyses. This integration of quantitative and qualitative evidence strengthened the study's mixed-methods design by enabling methodological triangulation and deeper interpretation of participants’ perceptions.

### Research instrument

The questionnaire was developed specifically for this study based on an extensive review of the environmental justice, sustainable development, environmental governance, and Sustainable Development Goals (SDGs) literature. The instrument was designed to assess citizens’ perceptions of environmental justice and the factors influencing its achievement in the West Bank.

The conceptual framework of the questionnaire was informed by the three core dimensions of environmental justice: distributive justice, procedural justice, and recognition justice. These dimensions were operationalized through sixteen SDG-based indicators representing environmental, social, economic, governance, and development-related domains relevant to the Palestinian context.

The questionnaire consists of 16 indicators aligned with key SDG-related domains, including:

1. Decent Work and Economic Growth (SDG 8)
2. Reduced Inequalities (SDG 10)
3. Sustainable Cities and Communities (SDG 11)
4. Responsible Consumption and Production (SDG 12)
5. Climate Action (SDG 13)
6. Life on Land (SDG 15)
7. Peace, Justice and Strong Institutions (SDG 16)
8. Partnerships for the Goals (SDG 17)

Each indicator includes multiple items measured using a three-point Likert scale, allowing respondents to express their perceptions regarding the extent to which environmental justice is achieved or obstructed within their communities.

The instrument specifically examines respondents’ perceptions of the role of occupation-related restrictions, governance challenges, institutional constraints, and development barriers in shaping environmental inequalities and sustainable development outcomes. The selected indicators were further used to assess perceived environmental justice conditions and their potential implications for environmental conditions and sustainable development in the West Bank.

To strengthen the conceptual linkage between environmental justice theory and the SDG-based indicators employed in this study, Table 1 presents the conceptual mapping between the selected SDG-based indicators and the three core dimensions of environmental justice: distributive justice, procedural justice, and recognition justice. The table illustrates how the selected indicators collectively capture the multidimensional nature of environmental justice and its relationship with sustainable development within the West Bank context. This mapping provides the conceptual foundation for the operationalization of the study variables and guides the interpretation of environmental justice across environmental, social, economic, governance, and development-related domains.

**Table 1:** Mapping of environmental justice items to SDG-based indicators

No	Item (Short Statement)	Indicator (Domain)	SDG	SDG Description
1	Obstruction of sustainable development plans	Global Partnership	SDG 17	Partnerships for the Goals
2	Limiting international institutional partnerships	Global Partnership	SDG 17	Partnerships for the Goals
3	Restricting local institutional cooperation	Institutions	SDG 16	Peace, Justice and Strong Institutions
4	Failure to fulfill development assistance commitments	Global Partnership	SDG 17	Partnerships for the Goals
5	Creation of digital divide	Inequalities	SDG 10	Reduced Inequalities
6	Constraints on economic opportunities	Economic Growth	SDG 8	Decent Work and Economic Growth
7	Inequality in access to resources	Inequalities	SDG 10	Reduced Inequalities
8	Restrictions on urban development and mobility	Sustainable Cities	SDG 11	Sustainable Cities and Communities
9	Weak environmental infrastructure	Consumption & Production	SDG 12	Responsible Consumption and Production
10	Increased pollution and environmental risks	Climate Action	SDG 13	Climate Action
11	Land degradation and biodiversity loss	Life on Land	SDG 15	Life on Land
12	Institutional and governance limitations	Institutions	SDG 16	Peace, Justice and Strong Institutions
13	Limited participation in decision-making	Institutions	SDG 16	Peace, Justice and Strong Institutions
14	Inefficiency in waste management systems	Consumption & Production	SDG 12	Responsible Consumption and Production
15	Constraints on climate adaptation	Climate Action	SDG 13	Climate Action
16	Weak global engagement	Global Partnership	SDG 17	Partnerships for the Goals

As shown in Table 1, distributive justice is reflected through indicators related to resource access, environmental quality, infrastructure, and development opportunities. Procedural justice is represented through indicators associated with participation, governance, institutional effectiveness, and decision-making processes, whereas recognition justice is reflected through indicators addressing inequalities, inclusion, and the differential impacts of environmental conditions on communities. The mapping demonstrates that environmental justice in the West Bank extends across multiple interconnected sustainability dimensions, including economic development (SDG 8), reduced inequalities (SDG 10), sustainable cities and communities (SDG 11), responsible consumption and production (SDG 12), climate action (SDG 13), life on land (SDG 15), peace, justice and strong institutions (SDG 16), and partnerships for the goals (SDG 17). The integration of environmental justice dimensions with SDG-based indicators provides the conceptual foundation for assessing environmental justice within the broader framework of sustainable development and supports the operationalization of the study variables.

### Validity and reliability

The questionnaire was developed following a comprehensive review of relevant literature on environmental justice, environmental governance, sustainable development, and SDG-based assessment frameworks. Content validity was established through a systematic review of the questionnaire items to ensure their relevance, clarity, and consistency with the study objectives and conceptual framework.

Prior to the main survey, a pilot study involving 35 participants was conducted to assess the clarity, comprehensibility, and appropriateness of the questionnaire items. Feedback obtained from the pilot participants was used to refine the instrument and improve item wording where necessary.

Internal consistency reliability was assessed using Cronbach’s alpha coefficient. The questionnaire consisted of 118 items distributed across sixteen SDG-based indicators. The overall Cronbach’s alpha coefficient for the instrument was 0.891, indicating a high level of reliability and internal consistency.

As shown in Table 2, the overall Cronbach's alpha coefficient for the study instrument was 0.891, indicating a high level of internal consistency and reliability. According to commonly accepted reliability standards, values exceeding 0.70 indicate acceptable reliability, while values above 0.80 indicate good reliability. Therefore, the obtained coefficient confirms that the questionnaire items consistently measured the underlying constructs of environmental justice and SDG-based dimensions, supporting the suitability of the instrument for subsequent statistical analyses.

**Table 2:** Presents the reliability statistics for the study instrument

Domain	Number of Items	Cronbach's Alpha
Total Instrument	118	0.891

### Data analysis

#### Quantitative analysis

Quantitative data were analyzed using statistical techniques to examine differences in perceptions across key variables: Gender, Place of residence (city, village, camp), Administrative division (north, central, south).

The following tests were applied:

1. Independent samples T-test to examine gender-based differences
2. One-way ANOVA to assess differences across residential and regional categories

A significance level of α ≤ 0.05 was used to determine statistical significance.

Descriptive statistics, including means and standard deviations, were calculated to evaluate the overall perception levels for each indicator. The resulting scores were used to assess respondents’ perceptions of environmental justice across the examined SDG-based dimensions and to explore their perceived implications for environmental conditions and sustainable development.

#### Qualitative analysis

The qualitative responses obtained from the open-ended section of the questionnaire were analyzed using a thematic analysis approach. Responses were systematically reviewed, coded, and categorized into recurring themes related to environmental inequalities, environmental governance, institutional limitations, barriers to sustainable development, and challenges associated with the implementation of the Sustainable Development Goals (SDGs).

The identified themes were subsequently used to contextualize and interpret the quantitative findings, providing additional insights into the perceptions of environmental justice among respondents and strengthening the overall mixed-methods design through methodological triangulation.

### Ethical considerations

Participation in the study was voluntary, and respondents were informed about the purpose of the research. Confidentiality and anonymity were ensured throughout the data collection and analysis process. The study adhered to ethical research standards, particularly in handling sensitive socio-political issues.

### Study limitations

While the study provides a comprehensive analysis, certain limitations should be acknowledged. The findings are based on perception-based data, which may be influenced by subjective experiences. However, the integration of qualitative insights helps mitigate this limitation by providing contextual depth.

Additionally, the complex political environment may impose constraints on data accessibility and generalization, although the sample size and spatial distribution strengthen the robustness of the results.

## Results

The results are presented according to the sixteen SDG-based environmental justice indicators. Descriptive statistics, including means and standard deviations, were used to assess the overall level of respondents’ perceptions. Independent-samples t-tests were used to examine gender-based differences, while one-way ANOVA was applied to examine differences according to place of residence and administrative division. Statistical significance was determined at *p* ≤ 0.05.

The item-level analysis presented in Table 3 indicates that all sixteen statements recorded high mean values, reflecting a strong and consistent perception among respondents regarding the presence of environmental injustice in the West Bank. The highest mean values were associated with restrictions on sustainable cities and inequalities, while slightly lower values were observed in indicators related to biodiversity. Nevertheless, all items remained within the high perception category, suggesting that environmental injustice is perceived as a comprehensive and multidimensional phenomenon affecting various aspects of life.

**Table 3:** Item-level results of environmental justice indicators

No	Item (Statement – Shortened)	Mean	Std. Deviation	Degree
1	Obstruction of sustainable development plans	2.908	0.290	High
2	Limiting international institutional partnerships	2.792	0.406	High
3	Restricting local institutional cooperation	2.731	0.444	High
4	Failure to fulfill development assistance commitments	2.893	0.309	High
5	Creation of digital divide between populations	2.816	0.388	High
6	Constraints on economic opportunities	2.868	0.178	High
7	Inequality in access to resources and services	2.908	0.156	High
8	Restrictions on urban development and mobility	2.932	0.122	High
9	Weakness in environmental infrastructure systems	2.724	0.193	High
10	Increased environmental pollution and risks	2.897	0.194	High
11	Degradation of land and biodiversity	2.405	0.175	High
12	Institutional and governance limitations	2.896	0.177	High
13	Limited participation in decision-making	2.884	0.182	High
14	Inefficiency in waste management systems	2.861	0.201	High
15	Constraints on climate adaptation measures	2.873	0.189	High
16	Weak global engagement in sustainable development	2.828	0.250	High

### Overall perceptions of environmental justice

**Fig. 2:**
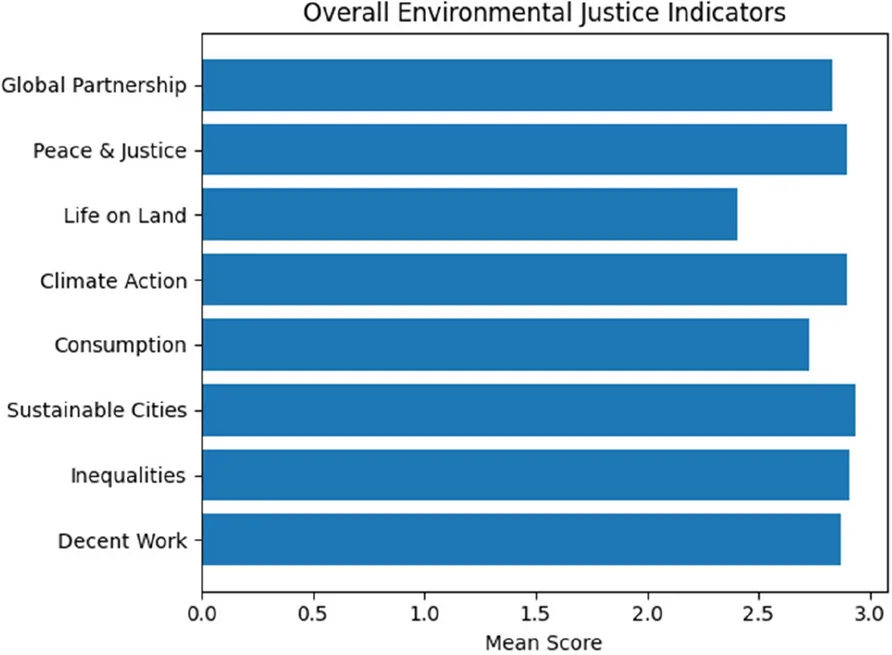
Overall mean scores of environmental justice indicators

The arithmetic means and standard deviations of the environmental justice indicators were analyzed to assess citizens’ perceptions of the state of environmental justice in the West Bank, Palestine, and the role of the Israeli occupation and its military laws in obstructing its achievement. Figure (4.2) presents the overall mean scores of the environmental justice indicators, while Table (4.3) provides the corresponding descriptive statistics, including the means and standard deviations for each indicator (Fig. 2).

#### Interpretation

Table 4 shows that all environmental justice indicators recorded high mean values, indicating a strong consensus among respondents regarding the existence of environmental inequalities in the West Bank. The highest mean was observed for Sustainable Cities and Communities (M = 2.932), followed by Reducing Inequalities (M = 2.908). The lowest mean was found in Life on Land (M = 2.405), although it still falls within the high perception category.

**Table 4:** Overall means and standard deviations of environmental justice indicators

Indicator	Mean	Std. Deviation	Degree
Decent Work and Economic Growth	2.868	0.178	High
Reducing Inequalities	2.908	0.156	High
Sustainable Cities and Communities	2.932	0.122	High
Responsible Consumption and Production	2.724	0.193	High
Climate Action	2.897	0.194	High
Life on Land (Biodiversity)	2.405	0.175	High
Peace, Justice and Strong Institutions	2.896	0.177	High
Global Partnership for Sustainable Development	2.828	0.250	High

This pattern suggests that environmental injustice is perceived as multidimensional and pervasive, affecting all sectors rather than being confined to a single domain.

### Differences by gender

#### Interpretation

Table 5 presents the results of the independent samples t-test examining gender differences in environmental justice indicators. The findings indicate that there are no statistically significant differences between male and female respondents across most indicators (p > 0.05), suggesting a high level of consistency in perceptions of environmental justice across gender groups.

**Table 5:** Independent samples T-test by gender

Indicator	Male Mean	Female Mean	T-value	Sig	Result
Decent Work	2.861	2.875	1.035	0.301	Not Significant
Inequalities	2.901	2.915	1.150	0.251	Not Significant
Sustainable Cities	2.933	2.930	0.305	0.627	Not Significant
Consumption	2.717	2.730	0.882	0.378	Not Significant
Climate Action	2.887	2.907	1.249	0.073	Not Significant
Life on Land	2.404	2.405	0.041	0.967	Not Significant
Peace & Justice	2.889	2.903	1.020	0.308	Not Significant
Global Partnership	2.806	2.852	2.308	0.021	Significant

However, a statistically significant difference was identified in the *Global Partnership for Sustainable Development* indicator (p = 0.021), with females reporting higher mean values compared to males.

**Fig. 3:**
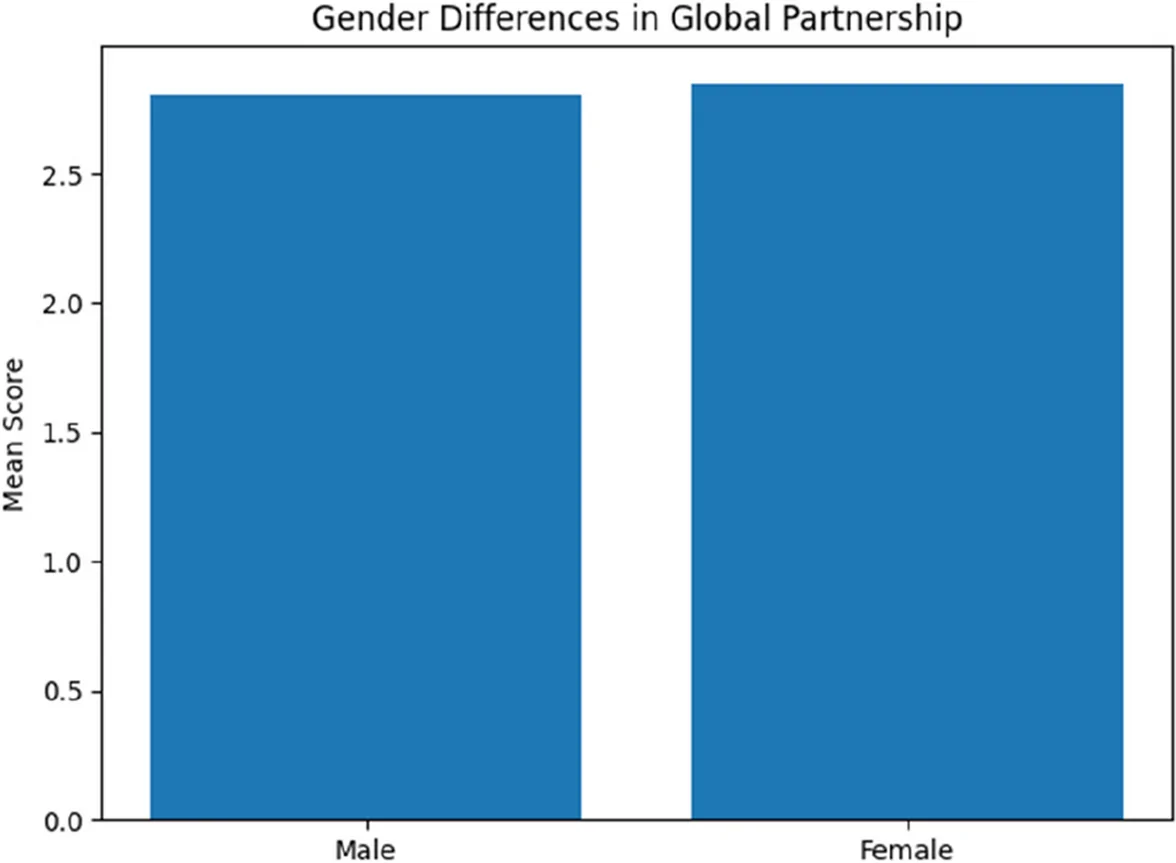
Gender differences in perceptions of global partnership for sustainable development. This visual difference, although moderate in magnitude, aligns with the statistical significance detected in the t-test results

This result is further illustrated in Fig. 3, which visually demonstrates the difference between male and female mean scores for this specific indicator. The figure highlights the relatively higher perception among female respondents, reinforcing the statistical significance observed in the t-test results.

This finding suggests that gender-based differences may emerge in dimensions related to international cooperation and institutional engagement, while remaining limited in structurally imposed environmental conditions.

### Differences by place of residence

Table 6 presents the mean values of environmental justice indicators across different residential settings (cities, villages, and refugee camps). The results show that the mean values are very close across all locations, with only minor variations. All indicators remain within the high perception range, indicating a generally consistent evaluation of environmental justice dimensions.

**Table 6:** Means by place of residence

Indicator	City	Village	Camp	Total
Decent Work	2.869	2.864	2.871	2.868
Inequalities	2.908	2.900	2.919	2.908
Sustainable Cities	2.940	2.930	2.924	2.932
Consumption	2.739	2.717	2.713	2.724
Climate Action	2.900	2.898	2.891	2.897
Life on Land	2.402	2.402	2.411	2.405
Peace & Justice	2.916	2.882	2.889	2.896
Global Partnership	2.836	2.822	2.826	2.828

**Fig. 4:**
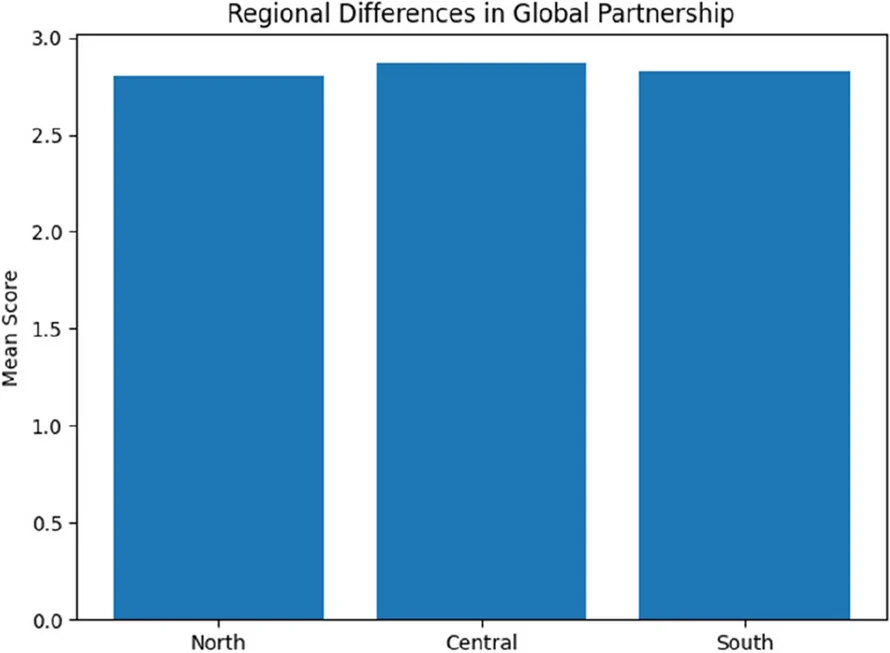
Regional differences in perceptions of global partnership for sustainable development

This pattern is visually illustrated in Fig. 4, which highlights the minimal differences between residential categories, particularly in the *Global Partnership for Sustainable Development* indicator. The figure demonstrates nearly overlapping mean values across cities, villages, and camps, reinforcing the absence of substantial spatial variation.

Furthermore, Table 7 confirms these observations through one-way ANOVA analysis, showing no statistically significant differences across all indicators (p > 0.05).

**Table 7:** One-way ANOVA by place of residence

Indicator	F-value	Sig	Result
Decent Work	0.096	0.908	Not Significant
Inequalities	0.703	0.496	Not Significant
Sustainable Cities	0.930	0.395	Not Significant
Consumption	1.072	0.343	Not Significant
Climate Action	0.134	0.874	Not Significant
Life on Land	0.177	0.837	Not Significant
Peace & Justice	2.323	0.099	Not Significant
Global Partnership	0.174	0.840	Not Significant

While Fig. 4 visually demonstrates minimal variation across residential categories, the ANOVA results (Table 7) confirm that these differences are not statistically significant.

#### Interpretation

Table 7 confirms that there are no statistically significant differences in perceptions of environmental justice based on place of residence (Sig > 0.05 for all indicators).

### Differences by administrative division

Table 8 presents the mean values of environmental justice indicators across administrative divisions (North, Central, and South). The results indicate that the mean values are highly similar across regions, with only minor variations observed. All indicators remain within the high perception range, reflecting a consistent evaluation of environmental justice dimensions across the study area.

**Table 8:** Means by administrative division

Indicator	North	Central	South	Total
Decent Work	2.869	2.882	2.855	2.868
Inequalities	2.907	2.900	2.915	2.908
Sustainable Cities	2.932	2.920	2.941	2.932
Consumption	2.724	2.736	2.714	2.724
Climate Action	2.889	2.906	2.900	2.897
Life on Land	2.409	2.410	2.396	2.405
Peace & Justice	2.885	2.903	2.904	2.896
Global Partnership	2.805	2.873	2.823	2.828

This pattern is visually illustrated in Fig. 4, which shows minimal variation between the three regions. The mean scores across the North, Central, and South appear closely aligned, with no substantial divergence across indicators.

These findings suggest that regional location has a limited influence on perceptions of environmental justice, indicating a broadly shared experience across administrative divisions.

#### Interpretation

Table 9 presents the results of the one-way ANOVA examining differences in environmental justice indicators across administrative divisions (North, Central, and South). The findings indicate that no statistically significant differences exist for most indicators (p > 0.05), suggesting a high level of consistency in perceptions across regions.

**Table 9:** One-way ANOVA by administrative division

Indicator	F-value	Sig	Result
Decent Work	1.072	0.343	Not Significant
Inequalities	0.428	0.652	Not Significant
Sustainable Cities	1.373	0.254	Not Significant
Consumption	0.564	0.569	Not Significant
Climate Action	0.416	0.660	Not Significant
Life on Land	0.430	0.651	Not Significant
Peace & Justice	0.815	0.443	Not Significant
Global Partnership	3.781	0.023	Significant

However, a statistically significant difference was identified in the Global Partnership for Sustainable Development indicator (p = 0.023).

**Fig. 5:**
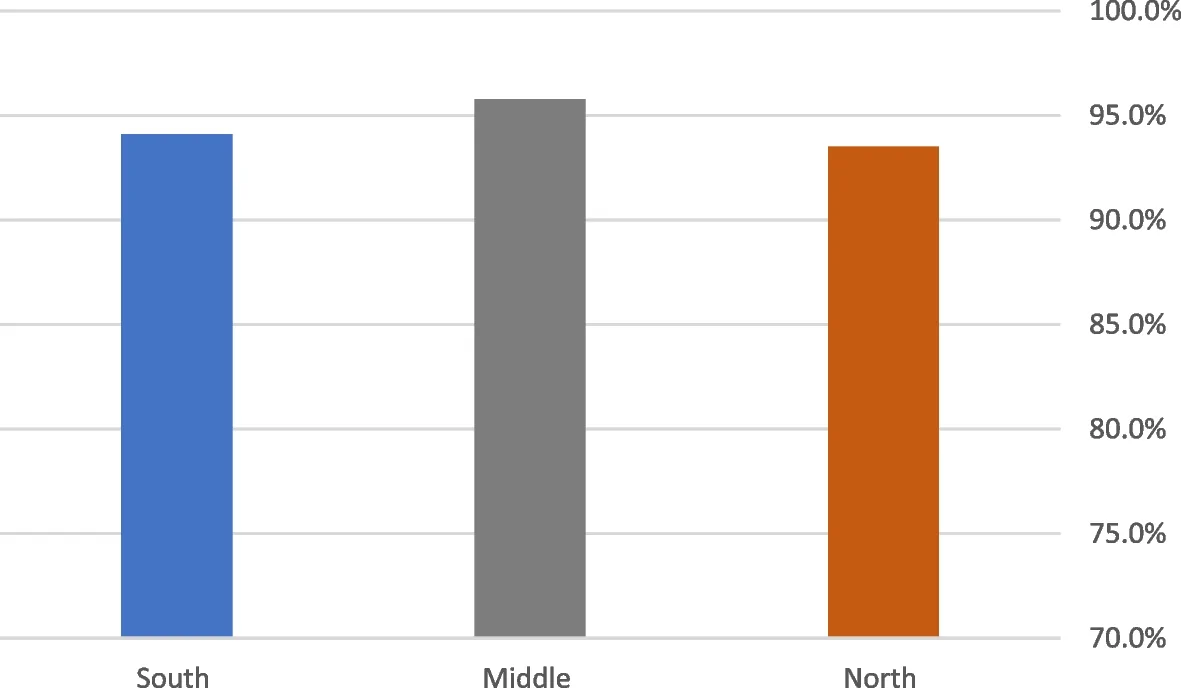
Perceptions of citizens regarding Global Partnership for Sustainable Development according to administrative division (North, Central, and South)

This result can be partially observed in Fig. 5, where slight variation appears between regions for this specific indicator, with the Central region showing relatively higher mean values compared to the North and South.

Despite this isolated difference, the overall pattern indicates that environmental justice perceptions are largely uniform across administrative divisions.

#### Interpretation

Following the identification of a statistically significant difference in the Global Partnership for Sustainable Development indicator through the ANOVA results (Table 9), the LSD post hoc test was conducted to determine the specific source of this variation.

As presented in Table 10, the results reveal that the significant difference is specifically between the North and Central regions (p < 0.05), with the Central region reporting higher mean values. In contrast, no statistically significant differences were found between the Central and South regions, nor between the North and South regions.

**Table 10:** LSD post Hoc test (Global partnership)

Comparison	Mean Difference	Significance
North vs Central	−0.0683	Significant
Central vs South	0.0499	Not Significant
North vs South	0.0183	Not Significant

These findings indicate that the overall significance observed in the ANOVA analysis is primarily driven by a localized difference rather than a consistent regional pattern across all administrative divisions.

Furthermore, this pattern suggests that the variation in perceptions of global partnership is linked to differential exposure to institutional and developmental contexts.

Therefore, the detected difference reflects a context-specific institutional dynamic rather than a broader geographically structured disparity.

### Summary of key findings

The results highlight three main patterns:

1. High Perception Levels Across All Indicators
  All SDG-based environmental justice indicators recorded high mean values, indicating strong perceptions of environmental justice concerns across multiple environmental, social, economic, governance, and development-related dimensions in the West Bank.
2. Broad Consistency Across Respondent Groups
  Most comparisons by gender, place of residence, and administrative division did not reveal statistically significant differences. This suggests that perceptions of environmental justice concerns were broadly similar across different demographic and geographical groups within the study sample.
3. Limited Variation in Global Partnership Perceptions
  The only indicator that showed statistically significant differences was Global Partnership for Sustainable Development. Significant differences were observed by gender and administrative division, indicating some variation in perceptions related to external cooperation and institutional engagement. However, the overall pattern remained characterized by high levels of perceived environmental justice concerns across all respondent groups.

## Discussion

### Environmental justice as a multidimensional and structural condition

The findings of this study are consistent with the conceptualization of environmental justice (EJ) as a multidimensional framework, as proposed by Schlosberg [25] and Walker [29]. The consistently high perception levels across all sixteen indicators reflect not only inequalities in environmental outcomes but also broader concerns related to governance, participation, and recognition.

In line with the literature, The results suggest that perceived environmental justice concerns in the West Bank extend across the three core EJ dimensions:

1. *Distributive justice*: evident in perceived inequalities in access to resources, infrastructure, and environmental services
2. *Procedural justice*: reflected in limited institutional capacity and restricted participation in environmental decision-making
3. *Recognition justice*: manifested in the marginalization of Palestinian communities and their limited control over environmental resources

This multidimensional pattern suggests that environmental justice concerns are closely associated with broader socio-political and institutional conditions.

### Consistency of perceptions and structural inequality

One of the most significant findings of this study is the absence of statistically significant differences across gender, place of residence, and administrative divisions for most indicators. This result suggests that perceptions of environmental justice concerns were broadly similar across respondent groups.

This finding aligns with studies such as Britto [8], Ferreira et al. [11], and Santos [24], which highlight that marginalized populations often experience environmental injustice due to systemic constraints rather than isolated factors. However, unlike many global contexts—where inequalities vary across socio-economic groups— the present study indicates a high degree of similarity in environmental justice perceptions across respondent groups, where injustice is perceived similarly across all segments of society.

These findings may reflect shared environmental and institutional conditions experienced across different geographical and demographic groups.

### Political ecology and the role of power

The results can be effectively interpreted through the lens of political ecology, which emphasizes the role of power, governance, and control over resources in shaping environmental outcomes. The high perception levels across indicators related to land use, infrastructure, and environmental management are consistent with findings by Rammal and El-Khazan [23] and the World Bank [31], which document how restrictions on land, water, and infrastructure development contribute to environmental inequality.

Furthermore, the qualitative findings reinforce this interpretation by highlighting how environmental challenges are closely linked to external control over resources and institutional limitations. This is consistent with interpretations that environmental justice concerns in the West Bank are influenced by political and institutional factors in addition to technical environmental challenges shaped by asymmetrical power relations.

Such findings are also consistent with Habbas [13], who describes the transformation of the West Bank into a space for environmental burden transfer, further illustrating how environmental inequalities are embedded within broader geopolitical dynamics.

### Environmental justice and development constraints

The study’s findings also resonate with broader literature linking environmental justice to development processes. As noted by Colmer et al. [9], environmental policies and economic systems often produce uneven outcomes that disproportionately affect vulnerable populations.

In the Palestinian context, these dynamics are further intensified by structural constraints on development, as highlighted by Alhumoud [3] and Al-Khatib et al. [5]. The high perception levels observed across indicators such as sustainable cities, climate action, and waste management reflect the cumulative impact of:

1. Limited infrastructure development
2. Weak institutional capacity
3. Financial and technical constraints

These findings confirm that environmental injustice in the West Bank is closely tied to developmental limitations, which are themselves shaped by political and structural conditions.

This interpretation is consistent with recent Palestinian scholarship highlighting the interconnections between resource scarcity, governance challenges, and sustainable development within the Palestinian context [1].

### Waste management as a critical dimension of environmental injustice

The results related to responsible consumption and environmental management can be directly linked to the literature on waste management challenges in Palestine. Studies by Lombrano [16], Sharholy et al. [27], and Pradhan [22] highlight the environmental and health risks associated with inadequate waste systems.

In the West Bank, these challenges are exacerbated by restrictions on infrastructure and land use, as documented by Alhumoud [3] and Al-Khatib et al. [5]. The findings of this study, which show high perception levels in this domain, reinforce the argument that waste management is not only a technical issue but also a justice-related concern, reflecting unequal exposure to environmental risks reflecting unequal exposure to environmental risks.

### Explaining the exception: global partnerships

The only indicator that showed statistically significant differences was global partnership for sustainable development, both across gender and administrative divisions. This finding can be interpreted in light of the literature on procedural justice and institutional engagement.

As noted by Armeni [6], barriers to participation and access to information can limit the ability of certain groups to engage with environmental governance processes. The higher perception levels among females may indicate differences in perceptions regarding institutional and international engagement to institutional and international dynamics, while regional differences.

This suggests that, unlike structurally imposed environmental conditions, institutional and external interactions are more variable, leading to differentiated perceptions.

### Implications for SDGs in conflict contexts

The findings of this study contribute to the growing body of literature questioning the applicability of global frameworks such as the SDGs in politically constrained environments. While the SDGs emphasize equity, sustainability, and inclusive development, the findings suggest that their implementation in the West Bank is significantly limited by structural and political barriers.

This aligns with previous research highlighting that development frameworks must be adapted to context-specific conditions, particularly in regions affected by conflict or external control. Without addressing these structural constraints, efforts to achieve sustainability may remain limited and uneven with significant implications for environmental health and human well-being.

### Toward a context-sensitive environmental justice framework

Overall, this study reinforces the need to reinterpret environmental justice within context-specific frameworks that account for political, institutional, and spatial dynamics. In the case of the West Bank, environmental justice cannot be fully understood without considering:

1. Structural constraints on resource access
2. Fragmented governance systems
3. External control over environmental and developmental processes

By integrating quantitative and qualitative findings, this study provides evidence that perceptions of environmental justice concerns in the West Bank are multidimensional and extend across multiple environmental, social, governance, and development-related domains, requiring approaches that go beyond conventional environmental and development paradigms.

This study suggests that environmental justice concerns in the West Bank extend beyond issues of environmental resource distribution and are closely associated with political, institutional, and governance-related conditions. The findings contribute to the environmental justice literature by illustrating how environmental justice perceptions may be shaped within a politically constrained context and by demonstrating the value of integrating SDG-based indicators into environmental justice assessment frameworks.

## Conclusion

This study examined environmental justice in the West Bank, Palestine, through a multidimensional framework informed by environmental justice theory and the Sustainable Development Goals (SDGs). By integrating quantitative data from 629 respondents with qualitative responses collected through open-ended survey questions, the study provides insights into how environmental justice concerns are perceived within a politically and institutionally constrained context.

The findings indicate high levels of perceived environmental justice concerns across all sixteen SDG-based indicators, suggesting that respondents identified environmental, social, economic, governance, and development-related challenges across multiple dimensions of environmental justice. Most indicators did not exhibit statistically significant differences according to gender, place of residence, or administrative division, indicating broadly similar perceptions across the study population.

The Global Partnership for Sustainable Development indicator represented the only dimension in which statistically significant differences were observed, suggesting some variation in perceptions related to institutional engagement and external cooperation. However, the overall pattern of results reflects a high degree of consistency in respondents’ perceptions across different demographic and geographical groups.

The study contributes to the environmental justice literature by applying an SDG-based assessment framework within the West Bank context and by illustrating how environmental justice concerns may be associated with political, institutional, and governance-related conditions. The findings further highlight the value of integrating environmental justice perspectives with sustainable development frameworks when examining environmental inequalities in politically constrained settings.

Overall, the results suggest that environmental justice concerns in the West Bank extend across multiple interconnected dimensions and should be understood within their broader environmental, social, institutional, and developmental context. Future research would benefit from incorporating objective environmental indicators, environmental monitoring data, and longitudinal approaches to further examine the relationships between environmental conditions, governance processes, and environmental justice outcomes.

### Policy implications

The findings of this study have important implications for policymakers, development agencies, and international organizations working in conflict-affected contexts.

First, the results highlight the need to reconsider the implementation of the SDGs in politically constrained environments. Development strategies should account for structural limitations related to governance, resource access, and institutional capacity, rather than assuming full local autonomy.

Second, environmental justice should be explicitly integrated into development planning by addressing its three core dimensions: distribution, participation, and recognition. This requires ensuring equitable access to environmental services, enhancing community participation in decision-making, and acknowledging the rights and needs of affected populations.

Third, there is a critical need to strengthen institutional capacity in environmental governance. This includes improving environmental monitoring systems, supporting local governance structures, and enhancing coordination across fragmented administrative regions.

Fourth, policies should prioritize improving infrastructure and environmental services, particularly in areas related to wastewater management, solid waste systems, and sustainable urban planning, while addressing disparities in service provision where they exist.

Finally, the study emphasizes the importance of enhancing international partnerships in a way that is context-sensitive and equity-oriented. Effective collaboration between Palestinian institutions and international organizations can support knowledge transfer, technical assistance, and resource mobilization, while addressing existing disparities.

This recommendation is particularly relevant given that the Global Partnership for Sustainable Development indicator was the only dimension that exhibited statistically significant variation across certain respondent groups, suggesting the importance of institutional engagement and external cooperation within the broader environmental justice framework.

### Limitations

Several limitations should be acknowledged when interpreting the findings of this study.

First, the study relied primarily on perception-based data collected through a structured questionnaire and qualitative interviews. While perceptions constitute an important dimension of environmental justice research and provide valuable insights into citizens’ experiences and perceptions, the study did not directly measure environmental exposures, pollution concentrations, water quality parameters, or health outcomes. Consequently, the findings should be interpreted as reflecting perceived environmental injustices and perceived environmental health risks rather than objectively measured environmental or health conditions.

Second, the use of a three-point Likert scale may have reduced response variability and contributed to potential ceiling effects, as many indicators achieved relatively high mean scores. Although this scale facilitated respondent participation and reduced response burden, future studies may benefit from using more sensitive measurement scales to capture greater variation in perceptions.

Third, although the qualitative component provided valuable contextual insights, future research could strengthen qualitative analysis through larger interview samples, more extensive thematic coding procedures, and greater integration between qualitative and quantitative findings.

Fourth, despite the relatively large sample size (*n* = 629) and its distribution across different regions and residential categories in the West Bank, there may be unobserved socio-economic, cultural, or contextual factors that influence perceptions of environmental justice but were not explicitly examined in this study.

Fifth, the study was conducted within a complex political, environmental, and institutional context that may limit the generalizability of the findings beyond the West Bank. Therefore, the results should be interpreted within the specific conditions of the occupied Palestinian territory and applied cautiously to other geographical settings.

Sixth, while respondents consistently identified occupation-related restrictions as important factors associated with environmental injustice, the study was not designed to isolate the independent causal effects of specific political or institutional policies. Accordingly, the findings indicate perceived associations rather than direct causal relationships.

Finally, the cross-sectional nature of the study limits the ability to assess temporal changes in environmental justice perceptions or to establish causal relationships over time. Future research integrating longitudinal data, environmental monitoring, spatial analysis, and objective environmental indicators would provide a more comprehensive understanding of environmental justice dynamics and their implications for sustainable development and environmental health.

### Future research

Building on the findings of this study, several directions for future research can be identified.

First, future studies could incorporate longitudinal data to examine how perceptions of environmental justice evolve over time, particularly in response to political or environmental changes.

Second, integrating spatial analysis tools such as GIS would allow for a more detailed examination of environmental inequalities and their geographical distribution.

Third, comparative studies across different regions—such as comparisons between the West Bank and Gaza Strip, or between Palestine and other conflict-affected regions—could provide broader insights into environmental justice under varying political conditions.

Fourth, future research could combine perception-based data with objective environmental indicators, such as water quality, pollution levels, and infrastructure availability, to provide a more comprehensive assessment.

Finally, further investigation into the role of international institutions and development programs could help clarify how external interventions influence environmental justice outcomes in politically constrained contexts.

## Supplementary Information

Supplementary Material 1.

Supplementary Material 2.

## Data Availability

Data Availability The datasets used and/or analyzed during the current study are available from the corresponding author on reasonable request.

## References

[1] Alayan S, Al-Natour A. Exploring the framings of water scarcity in palestinian textbooks. Contemporary Levant. 2023;8(1):3–15.

[2] Aldinger C. Environmental justice in Sub-Saharan Africa: Challenges and opportunities. J Environ Policy Plann. 2013;15(3):345–60.

[3] Alhumoud J. Municipal solid waste recycling in the Gulf countries. Resour Conserv Recycl. 2005;45(2):142–58.

[4] Al-Khatib IA, Arafat HA, Basheer T, Shawahneh H, Salahat A. Trends and problems of solid waste management in developing countries: A case study in Palestine. Waste Manag. 2007;27(12):1910–9. 17224264 10.1016/j.wasman.2006.11.006

[5] Al-Khatib IA, Monou M, Abu Zahra ASF, Shaheen HQ, Kassinos D. Solid waste characterization, quantification and management practices in developing countries: A case study from Palestine. J Environ Manag. 2010;91(5):1131–8. 10.1016/j.jenvman.2010.01.003 20116162

[6] Armeni C. Participation in environmental governance: Barriers and opportunities. Environ Law Rev. 2016;18(2):120–35.

[7] Bell D, Carrick J. Procedural environmental justice. Geography. Compass. 2017;11(12):e12359.

[8] Britto A. Environmental justice and urban inequality. Urban Stud. 2010;47(4):745–62.

[9] Colmer J, Hardman I, Shimshack J, Voorheis J. Disparities in PM2.5 air pollution in the United States. Science. 2020;369(6503):575–8. 32732425 10.1126/science.aaz9353

[10] Economic Commission for Latin America and the Caribbean (ECLAC). Regional Agreement on Access to Information, Public Participation and Justice in Environmental Matters in Latin America and the Caribbean (Escazú Agreement). 2018.

[11] Ferreira S, et al. Environmental inequality and human well-being. Ecol Econ. 2017;134:160–70.

[12] Franchini M, Mannucci L. Environmental justice and political participation. Environ Politics. 2018;27(6):1025–45.

[13] Habbas R. Environmental injustice in the occupied Palestinian territories. Middle East Policy. 2023;30(2):88–102.

[14] Human Rights Watch. A threshold crossed: Israeli authorities and the crimes of apartheid and persecution. 2021.

[15] Karasaki K, et al. Climate vulnerability and adaptive capacity in marginalized communities. Glob Environ Chang. 2023;78:102620.

[16] Lombrano A. Cost efficiency in the management of municipal solid waste. Waste Manag. 2009;29(7):2155–61.

[17] Martinez-Alier J, Temper L, Del Bene D, Scheidel A. Is there a global environmental justice movement? J Peasant Stud. 2016;43(3):731–55.

[18] Mbuligwe SE. Institutional challenges in solid waste management. Resour Conserv Recycl. 2002;35(3):131–46.

[19] McDonald DA. Environmental justice in South Africa. Geoforum. 2002;33(3):265–75.

[20] Mitchell G. Environmental justice in Europe: The role of governance. Environ Politics. 2019;28(2):295–316.

[21] Palestinian Central Bureau of Statistics (PCBS). Environment statistics report. 2010.

[22] Pradhan PK. Municipal solid waste management in developing countries. Waste Manag. 2008;28(2):303–11.

[23] Rammal M, El-Khazan H. Environmental governance under occupation: The case of Palestine. Environ Policy Gov. 2022;32(4):312–24.

[24] Santos B, de Sousa. Beyond abyssal thinking: from global lines to ecologies of knowledges. Review (Fernand Braudel Center). 2007;30(1):45-89.

[25] Schlosberg D. Defining environmental justice: Theories, movements, and nature. Oxford University Press; 2007.

[26] Schlosberg D, Collins LB. From environmental to climate justice. Wiley Interdiscip Rev Climate Change. 2014;5(3):359–74.

[27] Sharholy M, Ahmad K, Mahmood G, Trivedi RC. Municipal solid waste management in Indian cities. Waste Manag. 2008;28(2):459–67. 17433664 10.1016/j.wasman.2007.02.008

[28] United Nations Economic Commission for Europe (UNECE). Convention on Access to Information, Public Participation in Decision-Making and Access to Justice in Environmental Matters (Aarhus Convention). 1998.

[29] Walker G. Environmental justice: Concepts, evidence and politics. Routledge; 2012.

[30] Whyte K. Indigenous climate change studies: indigenizing futures. English Lang Notes. 2017;55(1–2):153–62.

[31] World Bank. Economic monitoring report to the Ad Hoc Liaison Committee. 2017.

