# Supplementary material for: Environmental injustice in the West Bank: a mixed-methods assessment using SDG-based indicators
Source: BMC Public Health. 2026 Jul 6;26:2576. doi: 10.1186/s12889-026-28228-w (PMC13560273; doi:10.1186/s12889-026-28228-w)
Supplement: Supplementary file 1 [file 12889_2026_28228_MOESM1_ESM.pdf]

Supplementary File 1: Questionnaire Used in the Study

Dear citizen,

Greetings.

The researcher is conducting a study entitled "Environmental Justice in the West Bank, Palestine, and the Role of the Israeli Occupation and its Military Laws in Obstructing or Preventing Its Achievement." This study is part of the requirements for a doctorate degree from the National Institute of Agricultural Sciences in Tunisia; to achieve the study's objectives, the researcher designed this questionnaire. We kindly ask you to answer it honestly, objectively, and accurately, as your response is important to obtaining the results. Please note that this information will be treated with complete confidentiality and is for scientific research purposes only.

Thank you for your cooperation in order to advance our beloved country.

Part I: Demographic Information

Please place an (X) in the box that best represents your status.

No.	Variable	Options
1	Gender	☐ Male ☐ Female
2	Place of Residence	☐ City ☐ Village ☐ Refugee Camp
3	Age	☐ Less than 20 ☐ 21–30 ☐ 31–40 ☐ 41 and above
4	Educational Level	☐ Secondary school or below ☐ Bachelor’s degree ☐ Master’s degree or higher
5	Occupation	....
6	Governorate (Region)	☐ Northern West Bank ☐ Central West Bank ☐ Southern West Bank

Part II: The questionnaire

No	Areas of environmental justice and their indicators:	Yes	Not sure	No
**Indicators of the first field: the reality of poverty.**				
1.	The occupation's practices have led to a significant increase in food prices.			
2.	The occupation has magnified the challenge facing Palestinians in combating poverty and food security.			
3.	The occupation has made unemployment almost permanent in Palestinian society.			
4.	The occupation's control of the crossings with neighboring countries has rendered most Palestinian youth unproductive in specialized business fields.			
5.	Israel is fighting Palestinian economic growth in all areas of development.			
6.	The practices of settlers and the occupation army have sparked a permanent conflict and devastating wars against the Palestinian people.			
7.	The occupation's practices prevent any system from achieving its goals of protection and social welfare.			
8.	The occupation prevents the creation of new employment prospects and job opportunities (travel bans or arrests).			
**Indicators for the second area: food security and hunger eradication.**				
9.	The occupation's restrictions prevent us from obtaining the necessary quantities of food for our children.			
10.	Palestinian food security is not achieved because the occupation prevents farmers from freely using all their land.			
11.	Palestinian food security is not achieved because the occupation prevents farmers from freely using their lands, which have been classified as A, B, and C.			
12.	Hunger and malnutrition are evident in Palestinian society due to the occupation preventing farmers from easily transporting produce to citizens.			

13.	Due to Israeli restrictions, it is difficult to meet my family's food needs.			
14.	Food prices are high due to taxes imposed on the transport of crops to Palestinian cities by the Israeli occupation.			
15.	Food production in the Palestinian territories is low due to the occupation's control over water resources.			
16.	The spread of Zionist settlements on Palestinian lands has contributed to hindering investment in the agricultural sector.			
17.	The occupation authorities prevent farmers from working freely in Palestinian and Arab markets.			
**Indicators of the third domain: good health and well-being.**				
18.	The number of child deaths has increased due to the occupation's prevention of the Palestinian Authority from importing the medical supplies it needs.			
19.	The occupation prevents citizens from traveling to Jerusalem hospitals, despite having health insurance or medical referrals.			
20.	The occupation controls the provision of Palestinian medical supplies, which has negatively impacted the level of health services provided to us.			
21.	The Israeli occupation does not provide various childhood vaccines and often prevents their entry from donor countries.			
22.	Israel prevents investment in various health systems and services in the Palestinian territories.			
23.	Restrictions imposed by the occupation have made it difficult for everyone to easily access vital healthcare services.			
24.	The level of awareness in Palestinian society of the importance of health education is weak due to the practices of the occupation.			
25.	Has water scarcity or poor water quality caused certain diseases in your area?			
26.	Has wastewater and improper treatment caused the spread of diseases in your area?			
27.	Have pollutants from factories located in settlements and their waste caused a climate and environmental disaster and diseases in the area where you live?			

28.	Do you believe that toxic gases emitted from Israeli military and industrial facilities are the reason behind the significant increase in the incidence of certain cancers?			
**Indicators of the fourth domain: quality education.**				
29.	The occupation's practices have prevented educational institutions from providing a high-quality education in a timely manner			
30.	Due to travel restrictions imposed on the Palestinian people, access to affordable vocational training is unavailable.			
31.	The occupation prevents the Palestinian Ministry of Education from achieving a high level of education.			
32.	The occupation imposes restrictions on education funding and programs supported by donor countries.			
33.	Restrictions imposed by the occupation on the movement of teachers and students to schools have made enrollment in education significantly weak in some places.			
34.	The occupation continues to practice a policy of demolishing schools and arresting teachers and students.			
35.	Continued efforts are being made to provide quality education for students, despite the occupation's policy of closing schools and firing toxic gases at students.			
36.	The occupation's policy has caused students and teachers to miss regular school hours.			
37.	The occupation also practices a heinous policy of impounding modern equipment at seaports for extended periods.			
**Indicators for the fifth area: gender equality and women's empowerment.**				
38.	Palestinian women working for some occupation institutions do not receive the same salaries as Israeli women.			
39.	The Israeli occupation fuels the violence in Palestinian society and the sexual exploitation of women.			
**Indicators for the sixth domain: clean water and sanitation services.**				
40.	The occupation prevents the Palestinian people from accessing their clean water needs.			
41.	The Israeli occupation controls all water resources in the West Bank.			

42.	The occupation's measures limit and reduce the level of services provided in the areas of sanitation and hygiene.			
43.	The amount of water provided to the settlements is significantly larger than the amount provided to Palestinian cities.			
44.	The occupation exploits Palestinian water resources and uses them in other sectors, such as agriculture, industry, and energy.			
45.	Due to the occupation, the Palestinian Water Authority is struggling to meet the growing demand for clean water for the Palestinian people.			
46.	The occupation's measures prevent investment in infrastructure and sanitation facilities in Palestinian cities.			
47.	The occupation's presence has limited the Palestinian Authority's ability to control and equitably distribute water resources across Palestinian cities.			
48.	The occupation's measures to prevent the construction of a sewage system have clearly led to problems related to the contamination of drinking water.			
**Indicators of the seventh area: Access to clean and affordable energy**.				
49.	The Palestinian people's access to electricity from the occupation has made their share of it scarce and expensive compared to the settlements.			
50.	The occupation imposes restrictions on investment in clean energy sources or importing them from neighboring countries.			
51.	The occupation monopolizes electricity generation in the Palestinian territories and distributes it at high prices.			
52.	The occupation prevents Palestinian society from accessing clean and safe fuels.			
53.	The energy prices provided by the occupation to the Palestinian people are very expensive compared to those of the settlers.			
54.	The occupation's restrictions have left many Palestinian villages without electricity day and night.			
55.	The Palestinian per capita share of electricity is much lower than that of the settlers.			

**Indicators for the eighth domain: Promoting inclusive and sustainable economic growth and providing employment and decent work opportunities for all.**				
56.	The occupation is creating difficult economic conditions for Palestinian society.			
57.	The occupation always attempts to form partnerships with all successful projects in the West Bank.			
58.	The occupation is the primary reason for the high Palestinian unemployment rate.			
59.	The occupation prevents the establishment of industrial cities in Palestinian cities, while allowing settlements to establish various factories.			
60.	The occupation's restrictions have made decent employment opportunities in Palestinian society virtually nonexistent.			
61.	The occupation combats all industrial projects competing with Israeli ones.			
62.	The occupation controls the import and export of Palestinian goods by imposing high taxes.			
63.	The occupation has also imposed high taxes on goods imported from Israeli ports.			
**Indicators for Domain 9: Decent Work and Economic Growth (Infrastructure).**				
64.	The occupation has struggled to establish an infrastructure capable of withstanding economic challenges.			
65.	The occupation only allows the construction of factories and competitive companies under prohibitive conditions.			
66.	The occupation holds back the necessary equipment for Palestinian companies at ports for extended periods.			
67.	The occupation raises the prices of energy needed by the economy to limit any Palestinian industrial or commercial progress.			
68.	The occupation blocks advanced and sophisticated generations of mobile communications devices while granting them to settlements.			
69.	The occupation prevents the Palestinian Authority from building new roads between cities without its approval.			
70.	The occupation clearly complicates trade with Arab countries.			

**Indicators for the tenth domain: Reducing inequalities.**				
71.	Settlers in the West Bank enjoy far greater opportunities, resources, and services than the Palestinian people.			
72.	The occupation's practices, particularly in marginalized areas, have created a large segment of the population in need of economic and educational support.			
73.	The occupation provides free transportation and education to residents of the settlements while denying them to the neighboring Palestinian people.			
74.	If the occupation of our homeland ends, we can achieve sustainable development and make our country a better place than it is now.			
75.	The occupation's continued aggression against the Palestinian people has created the problem of camps and internally displaced persons, causing chaos in the opportunities for equality and justice for all in all fields.			
76.	The occupation is fighting all Palestinian higher education institutions while opening all possibilities to Israeli universities in the settlements.			
77.	The occupation is fighting all international institutions that could contribute to achieving comprehensive social and economic growth.			
**Indicators of the eleventh domain: Sustainable cities.**				
78.	The occupation prevents the spread of safe Palestinian cities and prohibits the expansion of open spaces, granting them exclusively to settlers.			
79.	Due to the presence of Area C, as classified by the occupation, Palestinian cities suffer from significant overcrowding.			
80.	The presence of settlements has led to a lack of open public spaces, making it difficult for Palestinian cities to provide all the services they require.			
81.	Environmental pollution caused by settlements has contributed to the deterioration of everyone's health and impacted Palestinian farm production.			
82.	Military checkpoints and settler practices have made traffic between Palestinian cities complex and unsafe.			

83.	When comparing Palestinian cities with the settlements established on Palestinian land, we find a significant difference between them in terms of security, capabilities, and available opportunities.			
84.	The spread of settlements has led to a lack of security and safety for Palestinians inside and outside Palestinian cities and on the roads between them.			
**Indicators of the twelfth domain: responsible consumption and production.**				
85.	Comparing consumption habits between Palestinian citizens and residents of settlements is unfair in terms of quantity, price, or quality.			
86.	The occupation has made the energy supply of the Palestinian people less sustainable than that of the settlements.			
87.	The occupation's practices have contributed to low levels of commitment and awareness among the Palestinian people regarding consumption and production.			
88.	The occupation has contributed to environmental degradation, whether by preventing the establishment of nature reserves or by releasing and spreading wild animals on the outskirts of Palestinian cities.			
89.	The Israeli occupation's practices have limited the Palestinian Authority's ability to raise awareness and spread a culture of zero waste and the proper use of local resources.			
90.	Is what the Palestinian Authority is doing to raise awareness and spread a culture of zero waste and the proper use of local resources sufficient?			
91.	The occupation has completely subjugated the Palestinian economy.			
**Indicators for the thirteenth area: Climate action.**				
92.	The occupation's behavior regarding settlement sewage has contributed to the pollution of the Palestinian environment.			
93.	The occupation has created a climate catastrophe in the Palestinian territories by establishing factories within settlements that dump their waste in the vicinity of Palestinian cities.			
94.	The occupation has contributed to increased emissions of toxic gases from industrial facilities located in and near the West Bank, resulting in increased cancer cases among Palestinians.			

95.	The spread of Israeli industrial zones on the outskirts of settlements has contributed to the significant climate pollution in Palestinian cities.			
96.	The Israeli occupation is not taking immediate measures to avoid the disastrous consequences for Palestinian city residents.			
97.	Occupation laws prohibiting the transfer of waste to distant locations have contributed to climate pollution in the West Bank.			
**14. Indicators of the fourteenth domain: Life on land (biodiversity and desertification).**				
98.	The occupation does not contribute to the protection or restoration of terrestrial ecosystems, but rather works to destroy them, whether by building massive factories or by suppressing Palestinian agricultural projects.			
99.	The occupation authorities have encouraged the deterioration of green areas by encouraging work within the Green Line and the migration of Palestinian agricultural land.			
100.	The occupation has encouraged settlers to uproot or burn agricultural trees, especially olive and palm groves.			
101.	Despite the occupation's obstacles, Palestinian culture respects and preserves wildlife.			
102.	The occupation's classification of areas in the West Bank has hindered the establishment of areas as nature reserves, wilderness areas, or areas where construction is prohibited.			
103.	Are the Palestinian Authority's efforts to protect or restore wildlife ecosystems sufficient?			
104.	Does the Palestinian Authority provide protection for green areas?			
105.	Is what the Palestinian Authority is doing to protect agricultural trees, especially olive and palm groves, sufficient?			
106.	Is what the Palestinian Authority is doing to establish areas such as nature reserves, wilderness areas, or areas where construction is prohibited sufficient?			
**Indicators of the fifteenth domain: Peace, justice and strong institutions.**				
107.	The occupation has been keen to prevent all institutions from accessing information that achieves justice for all.			
108.	The occupation has fought to build effective and active institutions in Palestinian society.			

109.	The occupation's practices have placed Palestinian institutions in a state of constant fear of the occupation and its punishment of their employees.			
110.	The occupation's actions against employees of important Palestinian institutions have exposed them to danger and constant accountability.			
111.	The occupation has contributed to the flow of weapons into the hands of settlers and prevented them from reaching Palestinians.			
112.	The occupation contributes to and fuels the process of creating permanent problems and conflicts within Palestinian society.			
113.	The occupation does not allow freedom of expression to Palestinians, while granting it to settlers.			
**Indicators for the sixteenth area: Global partnership for sustainable development.**				
114.	The occupation governments have obstructed all practical plans to achieve sustainable development in Palestinian society.			
115.	The occupation has prevented the formation of sustainable and large-scale partnerships between Palestinian institutions and international organizations.			
116.	The Israeli occupation has prevented the formation of sustainable and large-scale partnerships between various Palestinian institutions.			
117.	The occupation government has not fulfilled its official development assistance commitments to the Palestinians.			
118.	The occupation has been keen to create a digital divide between Palestinians and settlers in internet use.			

Part III: Interview study questions;

1. How can the population of the West Bank, Palestine, be educated about their environmental rights?
2. Do residents of camps, cities, and villages in the West Bank, Palestine, enjoy the same environmental rights as residents of Israeli settlements and cities?
3. Are the capabilities of the Palestinian Environmental Authority sufficient to provide environmental conditions that meet the needs and international standards of the population?

4. Is the existence of Israeli occupation law, which controls everything from border crossings, water, air, and soil in the Palestinian territories, the reason for the failure to achieve environmental justice for Palestinians in the West Bank?
5. Does the Palestinian Authority, through the international community, exert pressure on the occupying power to achieve environmental justice for the Palestinian population in the West Bank?
6. Does the lack of environmental justice in the West Bank refugee camps and cities affect the mental health of Palestinian citizens?
7. Does overcrowding in Palestinian camps affect their mental and physical health?
8. Can the methods used to dispose of solid waste and wastewater in Palestinian camps be considered scientific and harmless to Palestinian citizens?

Thank you for your time and participation
